# Evaluation of the CL Detect Rapid Test in Ethiopian patients suspected for Cutaneous Leishmaniasis

**DOI:** 10.1371/journal.pntd.0010143

**Published:** 2022-01-18

**Authors:** Saskia van Henten, Helina Fikre, Roma Melkamu, Dilargachew Dessie, Tigist Mekonnen, Mekibib Kassa, Tadfe Bogale, Rezika Mohammed, Lieselotte Cnops, Florian Vogt, Myrthe Pareyn, Johan van Griensven

**Affiliations:** 1 Department of Clinical Sciences, Institute of Tropical Medicine, Antwerp, Belgium; 2 Leishmaniasis Research and Treatment Center, Gondar University Hospital, Gondar, Ethiopia; 3 National Centre for Epidemiology and Population Health, Research School of Population Health, College of Health and Medicine, Australian National University, Canberra, Australia; 4 The Kirby Institute, University of New South Wales, Sydney, Australia; KU Leuven, BELGIUM

## Abstract

**Background:**

Cutaneous leishmaniasis (CL) is common in Ethiopia, mainly affecting impoverished populations in rural areas with poor access to health care. CL is routinely diagnosed using skin slit smear microscopy, which requires skilled staff and appropriately equipped laboratories. We evaluated the CL Detect Rapid Test (InBios, Washington, USA), which is supplied with a dental broach sampling device, as a diagnostic alternative which could be used in field settings.

**Methodology/Principal findings:**

We evaluated the diagnostic accuracy of the CL Detect Rapid Test on skin slit and dental broach samples from suspected CL patients at the Leishmaniasis Research and Treatment Center in Gondar, Ethiopia. A combined reference test of microscopy and PCR on the skin slit sample was used, which was considered positive if one of the two tests was positive. We recruited 165 patients consecutively, of which 128 (77.6%) were confirmed as CL. All microscopy-positive results (n = 71) were also PCR-positive, and 57 patients were only positive for PCR. Sensitivity of the CL Detect Rapid Test on the skin slit was 31.3% (95% confidence interval (CI) 23.9–39.7), which was significantly higher (p = 0.010) than for the dental broach (22.7%, 95% CI 16.3–30.6). Sensitivity for both methods was significantly lower than for the routinely used microscopy, which had a sensitivity of 55.5% (IQR 46.8–63.8) compared to PCR as a reference.

**Conclusions/Significance:**

The diagnostic accuracy of the CL Detect Rapid Test was low for skin slit and dental broach samples. Therefore, we do not recommend its use neither in hospital nor field settings.

**Trial registration:**

This study is registered at ClinicalTrials.gov as NCT03837431.

## Author summary

Cutaneous leishmaniasis (CL) is common in Ethiopia, and mainly affects rural areas with poor access to health care. CL is routinely diagnosed using microscopy on a skin slit sample, which requires skilled staff and appropriately equipped laboratories. We evaluated the diagnostic accuracy of the CL Detect Rapid Test as an alternative which could be used in field settings. In a population of 165 patients suspected to have CL, 78% was confirmed to have CL by PCR. We found that the CL Detect Rapid Test on the supplied dental broach had a sensitivity of only 23%; on a skin slit sample, the Rapid Test had a slightly higher sensitivity with 31%. The routine diagnostic test of microscopy on the skin slit had a significantly higher sensitivity with 56%. These findings show that the sensitivity of the CL Detect Rapid Test is low, and is much worse than that of the currently used diagnostic method. Therefore, we do not recommend it to be used for diagnosing CL in Ethiopia.

## Introduction

Cutaneous leishmaniasis (CL) is a skin infection caused by intracellular protozoa that are transmitted by sand flies. It is endemic in many countries worldwide with an estimated yearly global incidence between 690,000 and 1,200,000 cases [1]. Different *Leishmania* species prevail in different geographical regions and differ by vector and reservoir. In Ethiopia, *L*. *aethiopica* is the main species causing CL, with *Phlebotomus pedifer* and *Phlebotomus longipes* acting as vectors, and hyraxes as their reservoir [2]. CL is common in rural areas of the Ethiopian highlands, where there is limited access to health care [3]. The estimated yearly incidence of CL in Ethiopia is between 20,000 and 50,000 cases [1], with children as an important patient group [4]. Most patients present to the hospital late, and only come if lesions do not heal on their own or do not respond to traditional treatment.

In Ethiopia, as in most resource-constrained CL-endemic settings, microscopic examination of a Giemsa-stained skin slit (synonyms are slit skin or skin scraping) smear is the standard method for diagnosing CL. It has excellent specificity and moderate sensitivity [5–8]. However, it requires substantial training for clinical and laboratory personnel as well as laboratories equipped with staining facilities and microscopes. Polymerase Chain Reaction (PCR) is more sensitive than microscopy [7,9–12], but is less suitable for routine diagnostic services in resource-limited settings due to costs and limited availability of consumables. Both microscopy and PCR usually rely on invasive sampling techniques such as skin slit or biopsy, although non-invasive swabs are increasingly used in Latin America. Decentralizing diagnosis of CL to lower health care settings may decrease patient delay and improve treatment outcomes, but existing CL diagnostics are unsuitable for decentralized health facilities. Thus, the majority of CL cases in low-resource settings remain undiagnosed and untreated.

A diagnostic test for CL which is sensitive, specific, rapid, simple, robust and can be implemented in resource-limited settings is urgently needed, while affordability also needs to be taken into account [13]. In 2014, the "CL Detect Rapid Test for Cutaneous Leishmaniasis" (InBios, Washington, USA) became available and received approval by the USA Food and Drug Administration after clinical testing in a region known to be endemic for *L*. *major*. The InBios CL Detect Rapid Test detects the peroxidoxin antigen produced by *Leishmania* amastigotes in skin lesions [14]. It is intended to be used on ulcerative lesions of less than four months duration and is supplied with a dental broach (for sample collection. The CL Detect Rapid Test is easy to read, relatively cheap and does not require advanced laboratory equipment. Thus, it may be suitable for field conditions and enable extension of CL care and treatment to rural areas in Ethiopia.

The CL Detect Rapid Test has been evaluated in various endemic settings with varying results [15–18]. However, it is not known how the test performs for *L*. *aethiopica*, and whether there is a difference in test performance for dental broach or skin slit samples. Therefore, we evaluated the diagnostic accuracy of the InBios CL Detect Rapid Test in a population of CL-suspected patients in Ethiopia using both skin slit and dental broach samples.

## Methods

### Ethics statement

This study was approved by the ethical review committees of the Institute of Tropical Medicine in Antwerp (1219/18), the University Hospital of Antwerp (18/08/085), and the University of Gondar (O/V/P/RCS/05/626/2019). Written informed consent was obtained from all participants or from the guardian/parent of patients below the age of 18. Assent was additionally collected for patients aged 12–17 years. This study is registered at ClinicalTrials.gov as NCT03837431. (https://clinicaltrials.gov/ct2/show/NCT03837431?cond=Leishmaniasis%2C+Cutaneous&draw=10&rank=15).

### Setting

The study was conducted at the Leishmaniasis Research and Treatment Center (LRTC) in Gondar, Ethiopia. This site serves as a referral center for CL patients in North-West Ethiopia. Patients with skin conditions are often first seen at the dermatology department, and referred to the LRTC for parasitological confirmation and further management when there is suspicion of CL. Microscopic examination of Giemsa-stained skin slits is the routine test used for confirmation of CL. If positive, slides are graded from +1 to +6 as recommended by the World Health Organization [19]. Microscopy slides were reviewed independently by two different readers.

### Design, population and recruitment

This was a cross-sectional diagnostic accuracy study among CL-suspected patients. The performance of the InBios CL Detect Rapid Test was assessed using a dental broach sample as well as a skin slit sample by comparing it to a combined reference test of skin slit microscopy and skin slit PCR. A patient was considered positive for the reference test if either skin slit microscopy or PCR was positive. Patients for whom the PCR result was invalid and microscopy was negative were not included in the diagnostic accuracy analysis. We followed the Standards for Reporting Diagnostic Accuracy (STARD) criteria for studies of diagnostic accuracy (S1 Table) [20].

CL-suspected patients were enrolled from February 2019 to December 2020 if they fulfilled the following criteria: age ≥2 years; CL lesion on suitable location for skin slit and dental broach sample (e.g. not on eyelid); not being on modern CL treatment and no comorbidity with visceral leishmaniasis (since the CL Detect Rapid Test also detects its causative agent *L*. *donovani*). Patients with all types of CL lesions (e.g. nodular, plaque etc.) and of all durations were included.

### Sample collection

Topical EMLA cream (5% lidocaine/prilocaine) was applied on the lesion for 30–60 minutes to limit patient discomfort. The dental broach (which is like a very thin needle with barbs for tissue retrieval, see S1 Fig) sample was taken according to the supplied instructions on the part of the lesion that was assumed to be most active (predominantly on the border, and avoiding mucosal parts of the lesion where possible) [14]. The sample was placed in three drops of kit lysis buffer, and tissue was flushed from the dental broach using a pipet, after which the dental broach was discarded. The sample was further processed after a 10 minute incubation for the CL Detect Rapid Test and PCR (see below). Furthermore, two skin slits were taken on approximately the same lesion site as the dental broach sample. The first skin slit was smeared on a microscopy slide and stained with Giemsa as per routine practice. Results were read by two readers blinded to the other readers’ microscopy result as well as the PCR results (complete blinding to index test results could not be guaranteed, as the same staff sometimes performed both tests), and positive results were rated from +1 to +6. The second skin slit was incubated for 10 minutes in a tube containing three drops of kit lysis buffer and tissue was flushed from the scalpel using a pipet, after which the scalpel was discarded.

### CL detect rapid test

20 μL of the dental broach or skin slit sample in lysis buffer was immediately added on the test strip, after which it was placed in a tube containing 3 drops of chase buffer. The test was read and checked (blinded to PCR and microscopy results) after 20 minutes. The remainder of the samples in lysis buffer were stored at -80°C after addition of one extra drop of lysis buffer until further processing for PCR.

### Molecular tests

DNA was extracted from the stored dental broach and skin slit on microscopy slides using the LEV blood DNA extraction kit (Promega, Leiden, The Netherlands) and the automated Maxwell 16 device (Promega). Kit lysis buffer was added, followed by proteinase K. This was vortexed and incubated at 56°C at 400 rpm for 20 minutes and loaded into the Maxwell device according to the manufacturer’s instructions. The eluted DNA extracts were stored at -80°C. In each batch of 15 samples, a negative extraction control (NEC, only lysis buffer) was included. *Leishmania* DNA was detected by a real-time PCR targeting the kDNA as described before [21] using the Rotor-Gene Q instrument (Qiagen, Venlo, The Netherlands). Positive and negative PCR controls and the NECs were included in each PCR run. Results were expressed in cycle threshold (Ct)-values. When the NEC was negative, the run was valid, and a sample was called positive for Ct values under 35 and repeated for Ct values >35. The latter were only called positive if a signal was detected again in the repeat run. If the negative extraction control was positive (any Ct), only samples with a Ct value at least 3 Ct values lower than the NEC were considered positive. Samples with a Ct value higher or less than 3 Ct values lower than the negative extraction control were repeated along with the NEC. After this, samples with a value higher or <3 Ct values lower than the negative extraction control were interpreted as invalid and excluded from the analyses. Samples that were negative (no signal after 50 cycles) for kDNA were subjected to a PCR targeting the human beta globin gene to monitor the extraction efficiency and PCR inhibition [22]. PCR tests were done in batch, blinded to index test results.

### Definitions

CL patients were classified into different CL types by the study physician according to the national guidelines [23]. Localized CL (LCL) lesions are few in number and can vary in size; they are restricted to the skin on the site of the sand fly bite on exposed body parts. Mucocutaneous leishmaniasis (MCL) involves the mucosa, and can develop due to sand fly bites on the mucosal borders of the nose or mouth, but can also be caused by contiguous spread from cutaneous lesions. Diffuse CL (DCL) starts with few papular or nodular lesions followed by a gradual dissemination of the infection leading to multiple papular, nodular and plaque lesions involving larger areas of the skin.

### Data collection and analysis

Data was collected on paper-based forms and entered into an electronic form designed with Kobo Toolbox [24].

Data analysis was done in R version 3.6.1. Numbers and proportions and medians and interquartile range (IQR) were used to describe the population. Sensitivity, specificity, as well as positive and negative predictive values with 95% confidence intervals were calculated for the InBios CL Detect Rapid Test using the dental broach and the skin slit samples, against a combined reference of skin slit microscopy and PCR.

Subgroup analyses were done for the different CL types, and for patients with ulcerative lesions of less than four months duration (which is the population as recommended by the InBios CL Detect Rapid Test manual) using Chi-square tests. McNemar’s test was used to compare the sensitivity of the CL Detect Rapid Test on the skin slit with the dental broach.

### Sample size

Our initial sample size calculation was done based on a precision of 10%, a power of 80% and an estimated sensitivity and specificity of the rapid test using the skin slit sample of 70%, which gave a sample size of 305 participants. After adding 15% due to expected loss to follow up, our initial sample size was 350 participants. Due to unavailability of the InBios CL Detect Rapid Test since June 2020, we had to stop recruitment early. We analyzed the 165 recruited patients for whom this test was done.

## Results

A total of 165 CL-suspected patients were included into this study, amongst 195 eligible patients (see S2 and S3 Figs for patient flowcharts). Their socio-demographic and clinical characteristics are described in Table 1. More than half (108; 65.5%) of the patients were male, with a median age of 22 years (IQR 18.0–38.0). The biggest group of patients was comprised of students (65; 39.4%), followed by farmers (30; 18.2%), and government employees (22, 13.3%). Most of the patients (100; 61.0%) live in rural areas, but interestingly, more than a quarter (47; 28.5%) of all patients came from Gondar town. The districts (called woredas in Ethiopia) where study participants originated from are shown in S3 Fig.

**Table 1:** Baseline characteristics.

Characteristic	Total (N = 165^a^)
Male sex, n (%)^b^	108 (65.5)
Age (years), median (IQR)	22.0 (18.0–38.0)
Occupation *(N = 164)*	
Student	65 (39.4)
Farmer	30 (18.2)
Government employer	22 (13.3)
Housewife	14 (8.5)
Other	33 (20.0)
Rural residence *(N = 164)*	100 (61.0)
Previous CL *(N = 164)*	20 (12.2)
Use of prior traditional treatment^c^	85 (51.5)
Lesion duration (months), median (IQR)	11.0 (6.0–18.0)
Nr of lesions, median (IQR) *(N = 163)*	1.0 (1.0–2.0)
Size of lesion^d^ (cm), median (IQR) *(N = 161)*	5.0 (3.0–10.0)
Location of index lesion, n (%) *(N = 163)*	
Face	153 (93.9)
Arms and legs	10 (6.1)
Type of CL *(N = 163)*	
LCL	94 (57.7)
MCL	58 (35.6)
DCL	11 (6.7)
Presentation of index lesion^e^, n (%) (*N = 163)*	
Crusted	94 (57.7)
Swollen	93 (57.1)
Erythema	90 (55.2)
Plaque	73 (44.8)
Ulcerated	66 (40.5)
Papular	44 (27.0)
Hyperpigmented	41 (25.2)
Nodular	32 (19.6)
Scaly	29 (17.8)
Hypopigmented	12 (7.4)
Superinfection	9 (5.5)
Microscopy	
Negative	94 (57.0)
Positive^f^	71 (43.0)
PCR results	
Positive	128 (77.6)
Negative	26 (15.8)
Invalid	11 (6.7)

LCL: localized cutaneous leishmaniasis, MCL: mucocutaneous leishmaniasis, DCL: diffuse cutaneous leishmaniasis, IQR: interquartile range, HIV: human immunodeficiency virus

^a^In case information was not available for all patients, the total number of patients with information for this variable is indicated with (*N = x)*

^b^All percentages are column percentages

^c^Among the 85 patients who used traditional treatment, 62 used herbal treatment, 14 unspecified traditional treatment, 6 holy water/mud/soil, 2 burning by hot metal, and 4 a combination of holy water/mud and herbal treatment.

^d^By largest diameter

^e^Lesions can have multiple presentations, therefore the sum of the different categories can be larger than the whole

^f^Parasite grading was +1 (n = 11), +2 (n = 16), +3 (n = 17), +4 (n = 8), +5 (n = 17) and +6 (n = 2)

**Fig 1:**
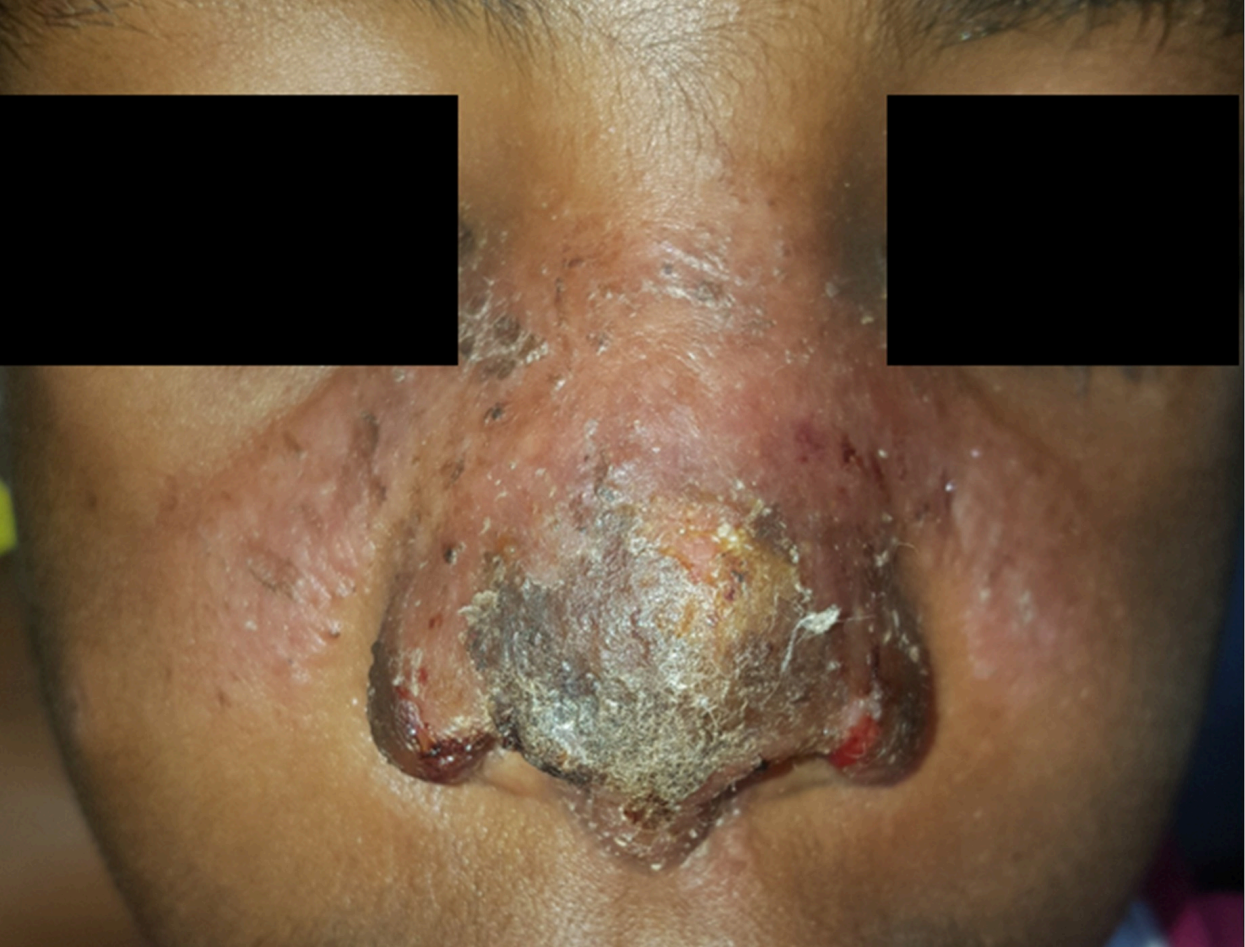
Image of a study patient. A typical presentation of CL, classified as MCL, with plaque, crust, swelling, erythema, and some ulceration as features. This patient was negative for the CL Detect Rapid Test on skin slit and dental broach, was positive for microscopy with grade +2, and positive for PCR with a Ct value of 22.0.

A total of 94 patients (57.7%) were classified as LCL, 58 (35.6%) as MCL, and 11 (6.7%) as DCL. The median duration of disease was 11 months (IQR 6.0–18.0 months), patients usually had one lesion (IQR 1.0–2.0) with a median size of 5.0 cm (IQR 3.0–10.0) and almost all (153; 93.9%) lesions were found on the face. Twenty patients reported having a previous CL episode, and more than a third (64; 38.8%) of the patients had used traditional treatment for their lesion in the past three months. Many different types of CL were seen, with crusts (94; 57.7%), swelling (93; 57.1%), erythema (90; 55.2%), and plaques (73; 44.8%) as common presentations. A total of 66 (40.5%) patients had lesions which had at least some ulceration (Table 1). A patient with a typical presentation is shown in Fig 1.

**Fig 2:**
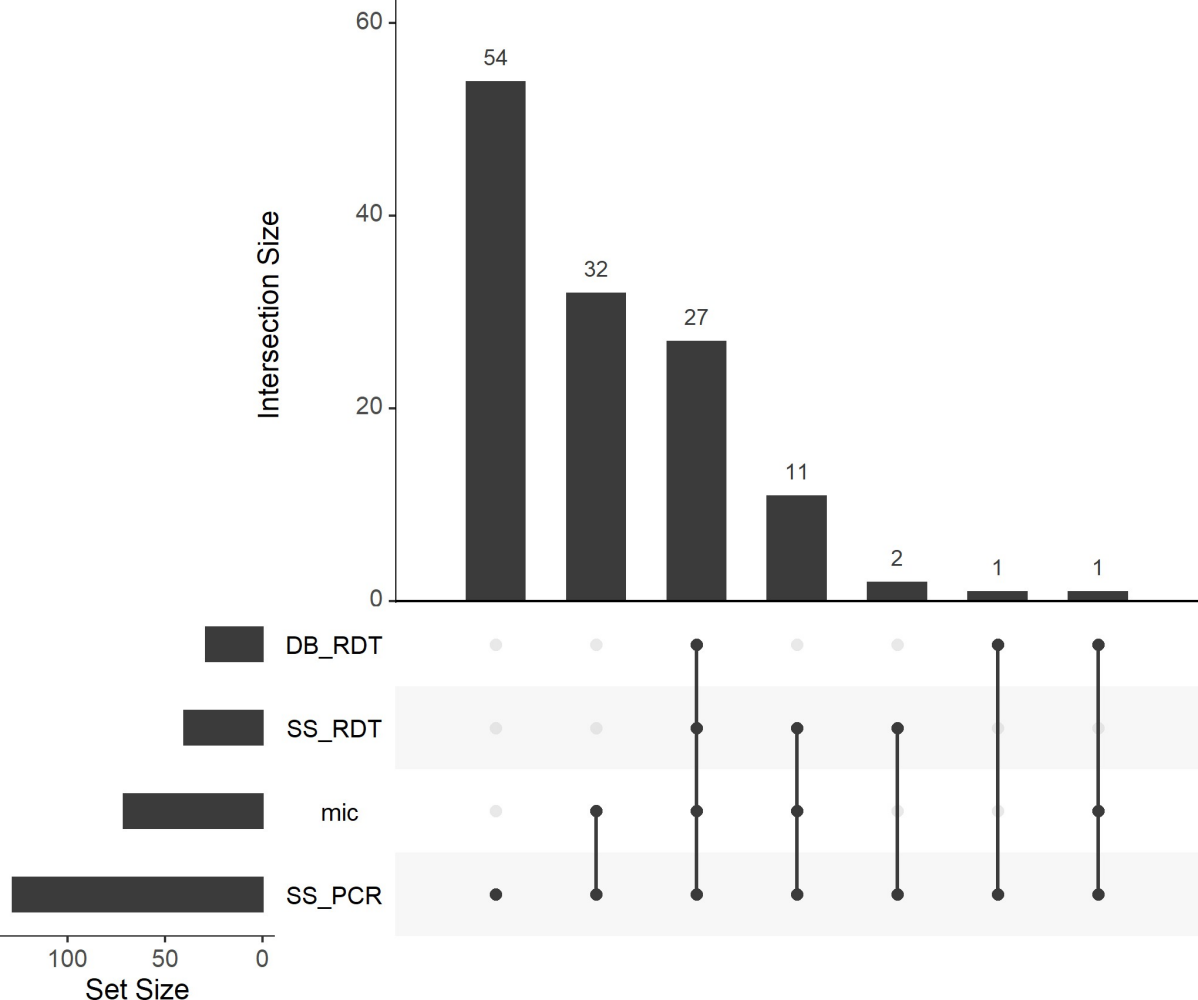
Overlapping test results for cutaneous leishmaniasis confirmed patients. This UpSet plot shows the overlap of the different diagnostic tests within the group of the 128 CL confirmed patients. The Intersection Size shows the number of patients positive for a certain selection of tests, which is indicated beneath the bars. The total amount of patients positive for each test is shown with the Set Size. DB: dental broach, SS: skin slit, RDT: CL Detect Rapid Test, mic: microscopy.

A diagram of the patient flow and results for the index and reference tests is shown in S2 Fig (skin slit) and S3 Fig (dental broach). In the study population, 128 patients (77.6%) were confirmed to have CL (positive on microscopy and/or PCR using a skin slit sample), 26 (15.8%) were negative for the combined reference test. All 128 confirmed patients were positive for PCR, whereas 71/128 (55.5%) were positive for microscopy as well as PCR, and 57/128 (44.5%) were negative for microscopy but positive by PCR. For 11 (6.7%) patients the true result of the reference could not be determined, as the PCR result was invalid, although microscopy was negative for all. Results for the different tests amongst the 128 confirmed patients are shown in Fig 2. A total of 54/128 (42.2%) patients were only positive for PCR and 32/128 (25.0%) were positive for both microscopy and PCR but not for other tests. Out of the confirmed patients 27/128 (21.1%) patients were positive for all tests. All confirmed patients who were positive for the CL Detect Rapid Test were also microscopy positive, except three patients who were only confirmed by PCR on skin slit.

Diagnostic accuracy for the CL Detect Rapid Test on the skin slit sample and the dental broach, as well as for the routinely used microscopy is shown in Table 2. The CL Detect Rapid Test was positive for 40 of the confirmed patients and 1 of the non-CL patients using the skin slit sample, and for 29 of the confirmed and one of the non-CL cases with the dental broach sample. The same non-CL case was positive for the CL Detect Rapid Test on both sample types. Compared to the combined reference of microscopy and PCR on skin slit, the sensitivity of the CL Detect Rapid Test using the skin slit sample was 31.3% (IQR 23.9–39.7), with a specificity of 96.2% (IQR 81.1–99.3). Sensitivity of the CL Detect Rapid Test using the dental broach was 22.7% (IQR 16.3–30.6), with the same specificity of 96.2% (81.1–99.3). The sensitivity of the CL Detect Rapid Test on the dental broach was significantly lower (p = 0.010) than that of the skin slit. Both tests had significantly lower sensitivity (p<0.001 for both) than the routinely used skin slit microscopy, which detected almost twice as many cases, and had a sensitivity of 55.5% (IQR 46.8–63.8) and a specificity 100% (IQR 87.1–100). The diagnostic accuracy did not change much if we interpreted the invalid PCR results as negative; this only decreased the 95% CI for specificity, and slightly improved the negative predictive value (S2 Table).

**Table 2:** Diagnostic performance of microscopy, the CL Detect Rapid Test on skin slit and dental broach samples.

	Cases, N = 128		Non-cases, N = 26		Diagnostic performance			
	Positive	Negative	Positive	Negative	Sensitivity (95% CI)	Specificity (95% CI)	PPV (95% CI)	NPV (95% CI)
Test								
Microscopy	71	57	0	26	55.5 (46.8–63.8)	100 (87.1–100)	100 (94.9–100)	31.3 (22.4–41.9)
SS RDT	40	88	1	25	31.3 (23.9–39.7)	96.2 (81.1–99.3)	97.6 (87.4–99.6)	22.1 (15.5–30.6)
DB RDT	29	99	1	25	22.7 (16.3–30.6)	96.2 (81.1–99.3)	96.7 (83.3–99.4)	20.2 (14.0–28.1)

CI: Confidence interval; DB: dental broach; RDT: Cl Detect Rapid Test, SS: Skin slit

Since the InBios CL Detect Rapid Test was specifically developed for ulcerative lesions of less than four months duration, we explored the performance of the tests according to duration of the lesion, and whether they were ulcerated or not. Only 16 confirmed patients had a lesion of less than 4 months duration (Table 3). In this group, sensitivity was still only 37.5% for both sample types (IQR 18.5–61.4). When we compared their sensitivity to those older than two years, the difference was not significant (p = 0.380), although the sensitivity in lesions of longer duration seems slightly lower with 16.7% for skin slits (IQR 6.7–35.9) and 12.5% for dental broaches (IQR 4.3–31.0).

**Table 3:** Sensitivity of the CL Detect Rapid Test by lesion duration.

	<4 months N = 16	4–11 months N = 54	12–23 months N = 34	≥24 months N = 24
Test	% (95% CI)	% (95% CI)	% (95% CI)	% (95% CI)
Skin slit RDT	37.5 (18.5–61.4)	35.2 (23.8–48.5)	32.4 (19.1–49.2)	16.7 (6.7–35.9)
Dental broach RDT	37.5 (18.5–61.4)	24.1 (14.6–36.9)	20.6 (10.3–37.0)	12.5 (4.3–31.0)

CI: Confidence interval; RDT: cl detect rapid test

Sensitivity was not significantly different (p = 0.085 for skin slit and 0.327 for dental broach) for ulcerated compared to non-ulcerated lesions (Table 4). In the 52 confirmed ulcerated cases, sensitivity was 28.9% for skin slit (IQR 18.3–42.3) and 17.3% for dental broach (IQR 9.4–29.7). In the 76 non-ulcerated patients, sensitivity was 32.2% for skin slit (95% CI 23.4–44.1) and 26.4% (95% CI 17.7–37.2) for dental broach.

**Table 4:** Sensitivity of the CL Detect Rapid Test by type of lesion.

	Ulcerated N = 52	Non-ulcerated N = 76
Test	% (95% CI)	% (95% CI)
Skin slit RDT	28.9 (18.3–42.3)	32.9 (23.4–44.1)
Dental broach RDT	17.3 (9.4–29.7)	26.3 (17.7–37.2)

CI: Confidence interval; RDT: InBios CL Detect Rapid Test

**Fig 3:**
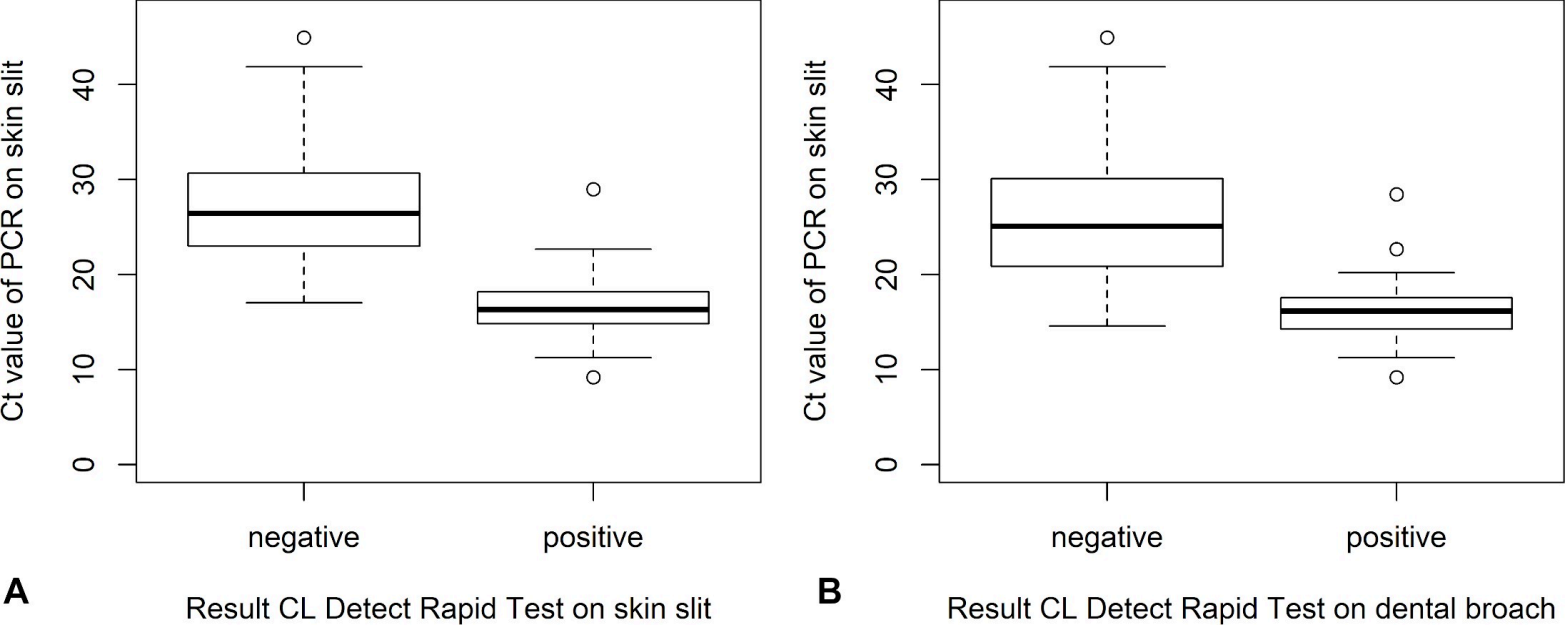
Ct values for patients positive versus negative on the CL Detect Rapid Test. The Ct values for the PCR on the skin slit sample are shown stratified by the result of the CL Detect Rapid Test, on the dental broach sample (A) and the skin slit sample (B). Ct values are significantly higher for patients who are negative for the CL Detect Rapid Test compared to those who are positive, regardless of the sample type.

Patients that were positive for the CL Detect Rapid Test using either a skin slit or a dental broach sample had significantly lower Ct values (Fig 3, p<0.001).

## Discussion

In this study we analyzed the diagnostic accuracy of the InBios CL Detect Rapid Test in a diverse cohort of 165 patients suspected to have CL in Ethiopia. The test showed poor sensitivity,. Results were significantly better when using a skin slit sample compared to the dental broach that is provided in the test kit. Results from subgroup analyses showed that test performance was not significantly better in patients with ulcerated lesions or lesions that were less than four months old. Specificity was good for both sample types and all kinds of lesions.

Other studies evaluating the InBios CL Detect Rapid Test showed rather conflicting results. Our findings are in line with research from Sri Lanka [18] and Suriname [15], where a sensitivity of around 36% was observed, although the specificity in Suriname was slightly lower at 84–86%. In Sri Lanka, *Leishmania donovani* is the causative species of CL, and in the study from Suriname, most lesions were due to *Leishmania guyanensis*. Results were better in Afghanistan (*L*. *tropica)* and Morocco (*L*. *tropica* and *L*. *major*), where the sensitivity was 65% [17] and 68% [16] respectively. Interestingly, in Morocco, where both *L*. *major* and *L*. *tropic* are endemic in different parts of the country, sensitivity was higher for *L*. *tropica* (73%, 95% CI 66–80), than for *L*. *major* (59%, 95% CI 47–70). The best sensitivity results were obtained in the clinical testing of the product by the producer in Tunisia (100%), in an area that is known to be endemic for *L*. *major* [14]. It should be noted that in that study microscopy was used as a reference, which is not adequately sensitive on its own to be used as a reference test.

Several reasons for poor sensitivity of the CL Detect Rapid Test have been mentioned in previous studies, including low parasite loads, and a lower expression of peroxidoxin (the target for the test) in certain species. Our results clearly show that the CL Detect Rapid Test is only able to pick up patients with lower Ct values, while in our population we observed many patients with a relatively high Ct value, indicating low parasite loads. The amino acid sequence of peroxidoxin in *L*. *aethiopica* was shown to be 94% (peroxidoxin2) and 91% (peroxidoxin1) identical to that of *L*. *major* [25], but the relative expression of peroxidoxin in *L*. *aethiopica* compared to *L*. *major* is unknown. Both a relatively low peroxidoxin expression and a mutation affecting the target recognition could have contributed to the low sensitivity observed. Another important factor that could account for variation in terms of sensitivity is the reference test used. We used the very sensitive kDNA PCR, which targets kinetoplast DNA minicircles which are present in 10,000–20,000 copies per cell, while most studies used the ITS-1 or 18S rRNA targets, which have a much lower copy number, making them less sensitive. A bigger problem in some studies is the use of microscopy as a reference. This could lead to wrongful classification of patients with very low parasite numbers as non-cases, and hence inflate the sensitivity of the InBios CL Detect Rapid Test.

A recent consensus document [13] defined that a point-of-care test for CL should have a sensitivity of at least 95% in parasitologically confirmed patients. However, our results show that the estimated sensitivity of the CL Detect Rapid test is only 22.7% for dental broach and 31.3% for skin slit, with 95% confidence intervals that do not surpass 40%. Based on the limited sensitivity, we do not recommend the use of the InBios CL Detect Rapid Test for routine use for the detection of *L*. *aethiopica*. Sensitivity of the test is only around half as high as microscopy, and although results for the InBios CL Detect Rapid Test are available within 25 minutes whereas microscopy can take up to an hour, this test still requires skilled staff to take the skin samples. Furthermore, the cost of 4 dollars per rapid test (which is still a reduced cost for research purposes) makes it significantly more expensive than microscopy. Lastly, availability of the rapid test has been interrupted since June 2020, which has forced us to prematurely stop the study as our previously ordered test kits had reached their two year expiry date. Optimizing the test procedure by using skin slits instead of dental broach samples, or by using it only on ulcerated or very young lesions also does not seem to improve the sensitivity of the test to levels required for routine use.

Our results showed that all patients with a positive microscopy test were confirmed with PCR, and that microscopy did not detect additional patients compared to PCR. This indicates that microscopy does not have additional value compared to PCR in this setting. Although PCR is not routinely available in hospitals in Ethiopia, our findings clearly demonstrate its superiority to microscopy. Molecular methods which are quicker, cheaper and easier to perform such as Loop-Mediated Isothermal Amplification assays may be suitable for routine use in resource-limited settings and should be explored for Ethiopia.

Although the majority of patients in our study come from rural areas, we were surprised to find many patients coming from Gondar Town. A previous study also showed that parts of Addis Ababa are endemic for CL [26]. Studies investigating the presence of *Leishmania* vectors in (semi)-urban areas in the Gondar area are currently ongoing and could serve as a starting point for interventions targeting transmission.

Strengths of this study are the fact that we tested the rapid test in a routine care setting among a representative population of suspected CL patients; that we used a very sensitive kDNA PCR as a reference test on the same sample used for the CL Detect Rapid Test; and that we used strict criteria to call a reference test positive or negative. However, this study is subject to several limitations. Since we encountered some contamination during DNA isolation, the PCR result was invalid for 11 samples as the sample values were too close to the value obtained for the contaminated negative extraction control. These samples are currently not included in the sensitivity and specificity analysis. If they were positive, this would lead to lowered sensitivity of the rapid tests, as all rapid tests (and microscopy results) of these patients were negative. If any of these cases were truly negatives, which is the most likely scenario, this does not affect the specificity estimates, although it causes the 95% confidence interval for these estimates to narrow. Another limitation is the relatively low number of negative patients, which means our specificity estimates are relatively inaccurate. Lastly, species typing is not included in this paper, but based on previous studies we expect all patients to be affected by *L*. *aethiopica* [8,27,28]. This will be verified in a follow-up study.

## Conclusion

We evaluated the diagnostic accuracy of the InBios CL Detect Rapid Test in CL-suspected patients in Ethiopia. Sensitivity was low for lesions of all durations and all types, and the sensitivity for the test using the dental broach was significantly lower than when using a skin slit sample. We do not recommend the use of the test in routine care, although the need for point of care tests in Ethiopia remains high.

## Supporting information

S1 Fig Dental broach sampling device. (TIF)

S2 Fig Flow diagram using the CL Detect Rapid Test on the skin slit as the index test. Reference test is a combined reference of PCR on a skin slit sample and microscopy on a skin slit sample. RDT:CL Detect Rapid Test.
(TIF)

S3 Fig Flow diagram using the CL Detect Rapid Test on the dental broach slit as the index test. Reference test is a combined reference of PCR on a skin slit sample and microscopy on a skin slit sample. RDT:CL Detect Rapid Test.
(TIF)

S4 Fig Map of districts where patients came from. This map shows Amhara and Addis Ababa region. Darker colors indicate more patients who came from a district. The number of patients coming from each district is indicated with a number. The University of Gondar Hospital is indicated with a white triangle. [29] source: https://data.humdata.org/dataset/ethiopia-cod-ab?.
(TIF)

S1 Table STARD checklist. (DOCX)

S2 Table Sensitivity analysis for the different diagnostic tests with re-classification of invalid test results. CI: Confidence interval; DB: dental broach; RDT: Cl Detect Rapid Test, SS: Skin slit.
(DOCX)
