# Supplementary figures and images for: Evaluation of the CL Detect Rapid Test in Ethiopian patients suspected for Cutaneous Leishmaniasis
Source: PLoS Negl Trop Dis. 2022 Jan 18;16(1):e0010143. doi: 10.1371/journal.pntd.0010143 (PMC8797207; doi:10.1371/journal.pntd.0010143)

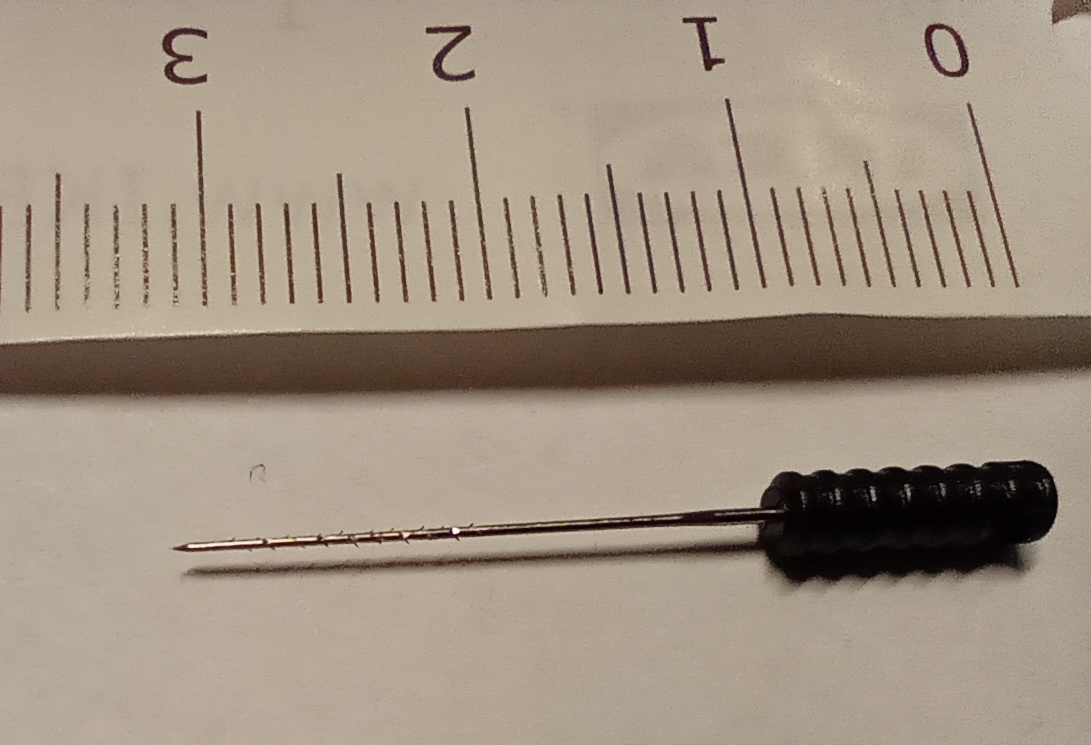

Supplement: S1 Fig — (TIF) [file pntd.0010143.s001.tif]

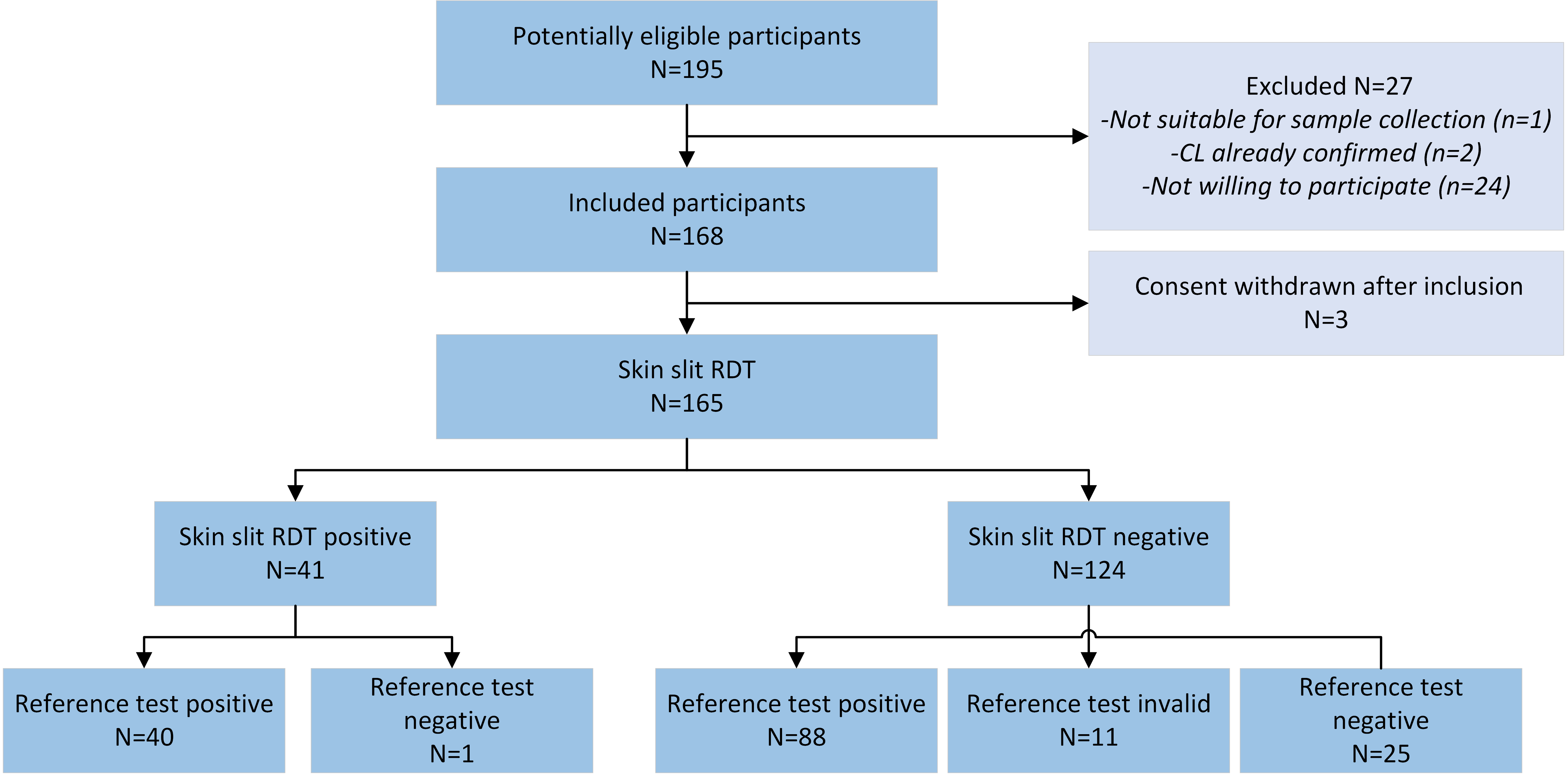

Supplement: S2 Fig — Reference test is a combined reference of PCR on a skin slit sample and microscopy on a skin slit sample. RDT:CL Detect Rapid Test. (TIF) [file pntd.0010143.s002.tif]

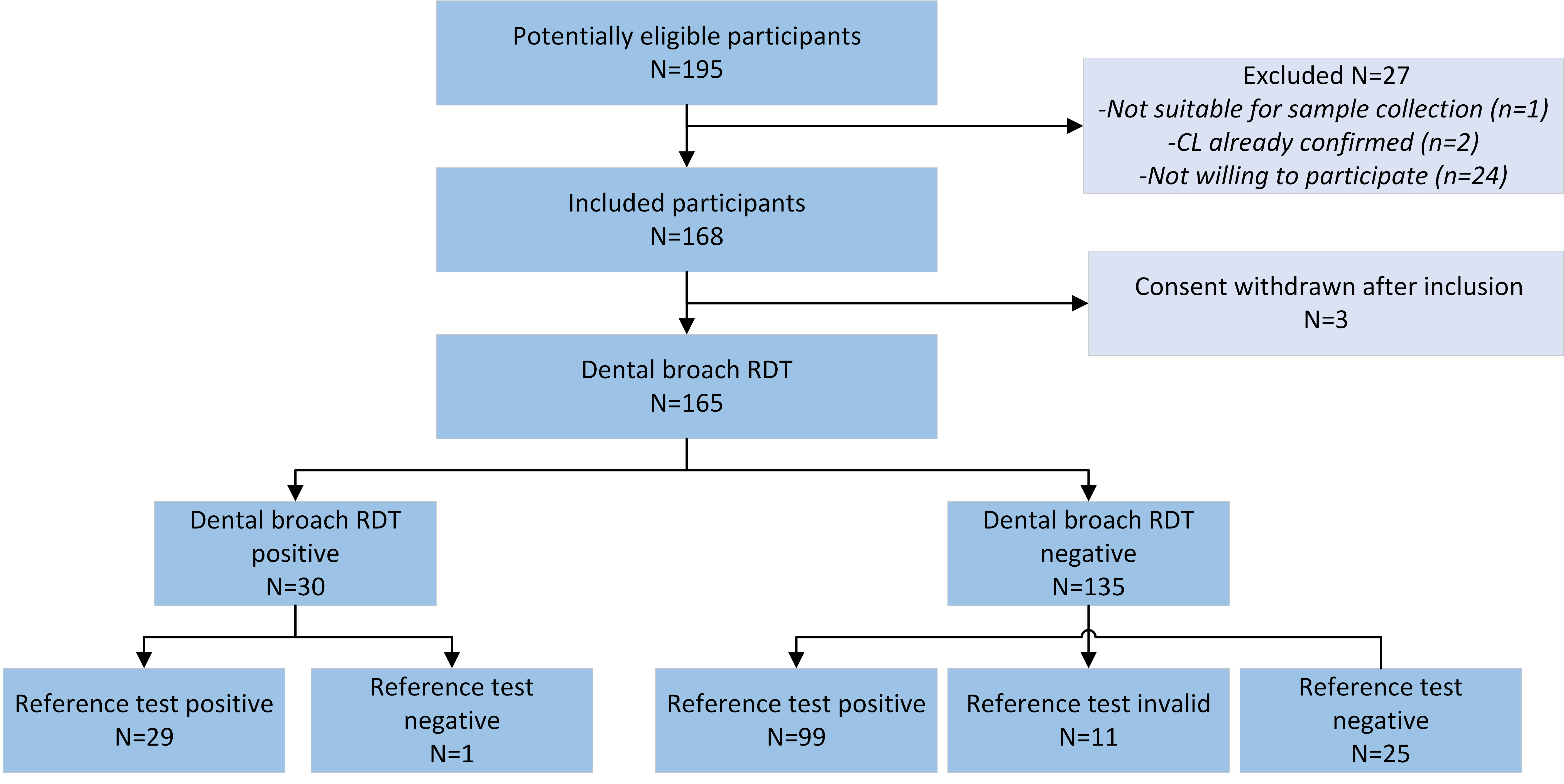

Supplement: S3 Fig — Reference test is a combined reference of PCR on a skin slit sample and microscopy on a skin slit sample. RDT:CL Detect Rapid Test. (TIF) [file pntd.0010143.s003.tif]

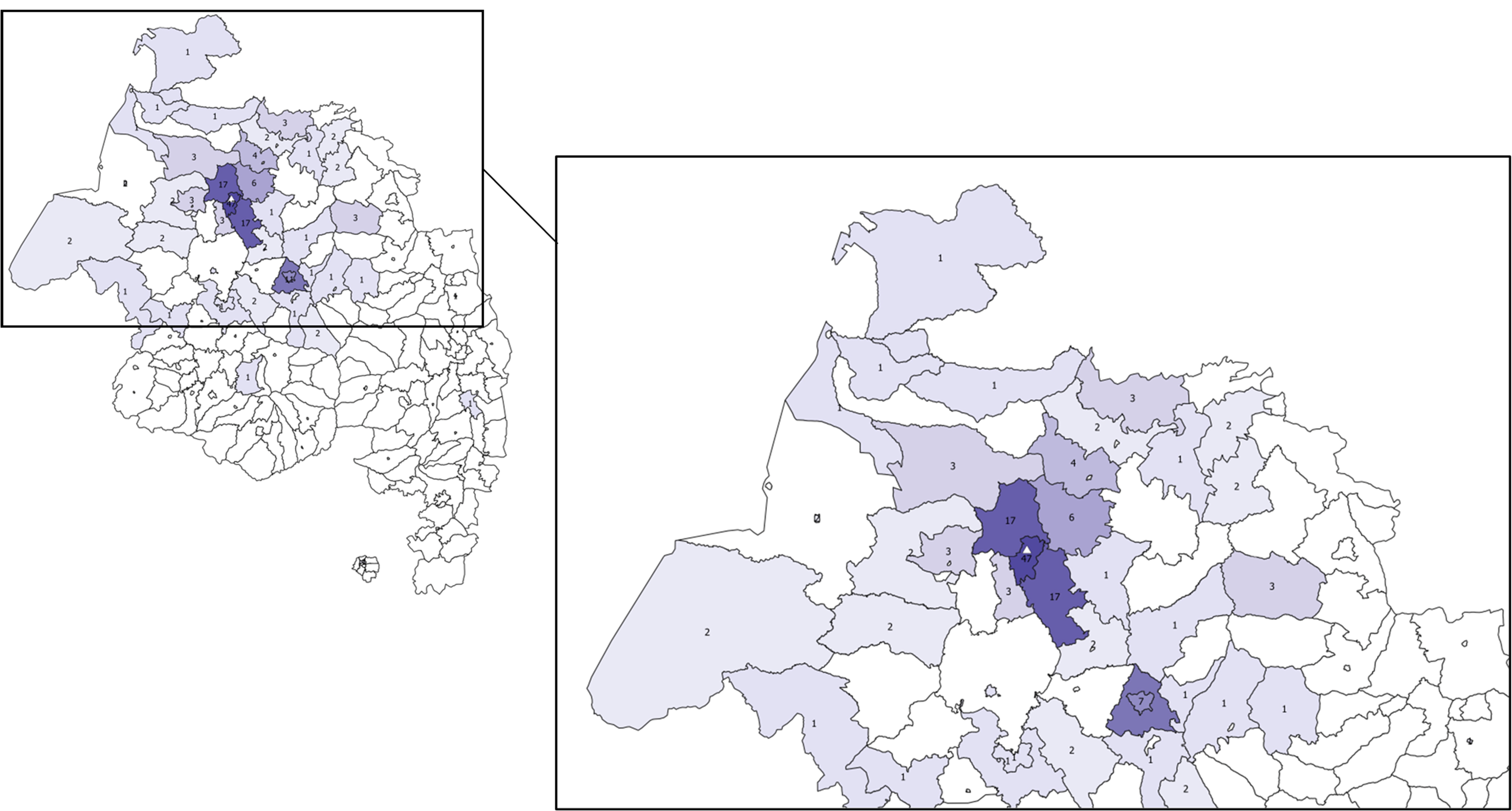

Supplement: S4 Fig — This map shows Amhara and Addis Ababa region. Darker colors indicate more patients who came from a district. The number of patients coming from each district is indicated with a number. The University of Gondar Hospital is indicated with a white triangle. [29] source: https://data.humdata.org/dataset/ethiopia-cod-ab?. (TIF) [file pntd.0010143.s004.tif]
