# Supplementary material for: Evaluation of the CL Detect Rapid Test in Ethiopian patients suspected for Cutaneous Leishmaniasis
Source: PLoS Negl Trop Dis. 2022 Jan 18;16(1):e0010143. doi: 10.1371/journal.pntd.0010143 (PMC8797207; doi:10.1371/journal.pntd.0010143)
Supplement: S1 Table — (DOCX) [file pntd.0010143.s005.docx]

|  | **Section & Topic** | **No** | **Item** | **Reported on page #** |
| --- | --- | --- | --- | --- |
|  |  |  |  |  |
|  | **TITLE OR ABSTRACT** |  |  |  |
|  |  | **1** | Identification as a study of diagnostic accuracy using at least one measure of accuracy  (such as sensitivity, specificity, predictive values, or AUC) | Abstract: *"We evaluated the diagnostic accuracy of the CL Detect Rapid Test"* |
|  | **ABSTRACT** |  |  |  |
|  |  | **2** | Structured summary of study design, methods, results, and conclusions  (for specific guidance, see STARD for Abstracts) | See abstract |
|  | **INTRODUCTION** |  |  |  |
|  |  | **3** | Scientific and clinical background, including the intended use and clinical role of the index test | *"The CL Detect Rapid Test is easy to read, relatively cheap and does not require advanced laboratory equipment. Thus, it may be suitable for field conditions and enable extension of CL care and treatment to rural areas in Ethiopia.*  *The CL Detect Rapid Test has been evaluated in various endemic settings with varying results [15–18]. However, it is not known how the test performs for L. aethiopica, and whether there is a difference in test performance for dental broach or skin slit samples. Therefore, we evaluated the diagnostic accuracy of the InBios CL Detect Rapid Test in a population of CL-suspected patients in Ethiopia using both skin slit and dental broach samples. "* |
|  |  | **4** | Study objectives and hypotheses | "The CL Detect Rapid Test has been evaluated in various endemic settings with varying results [15–18]. However, it is not known how the test performs for L. aethiopica, and whether there is a difference in test performance for dental broach or skin slit samples. Therefore, we evaluated the diagnostic accuracy of the InBios CL Detect Rapid Test in a population of CL-suspected patients in Ethiopia using both skin slit and dental broach samples. " |
|  | **METHODS** |  |  |  |
|  | *Study design* | **5** | Whether data collection was planned before the index test and reference standard  were performed (prospective study) or after (retrospective study) | Methods section, subsection Design, population and recruitment: *"CL-suspected patients were enrolled consecutively from February 2019 to December 2020"* |
|  | *Participants* | **6** | Eligibility criteria | Methods section, subsection Design, population and recruitment: "*CL-suspected patients were enrolled […] if they fulfilled the following criteria: age >2 years; CL lesion on suitable location for skin slit and dental broach sample (e.g. not on eyelid); not being on modern CL treatment and no comorbidity with visceral leishmaniasis (since the CL detect Rapid Test also detects its causative agent L. donovani). Patients with all types of CL lesions (e.g. nodular, plaque etc.) and of all durations were included."* |
|  |  | **7** | On what basis potentially eligible participants were identified  (such as symptoms, results from previous tests, inclusion in registry) | Methods section, subsection Setting: *"Patients with skin conditions are often first seen at the dermatology department, and referred to the LRTC for parasitological confirmation and further management when there is suspicion of CL."* |
|  |  | **8** | Where and when potentially eligible participants were identified (setting, location and dates) | Methods section, subsection Setting: *"Patients with skin conditions are often first seen at the dermatology department, and referred to the LRTC for parasitological confirmation and further management when there is suspicion of CL."*  Methods section, subsection Design, population and recruitment: *"CL-suspected patients were enrolled from February 2019 to December 2020:"* |
|  |  | **9** | Whether participants formed a consecutive, random or convenience series | Methods section, subsection Design, population and recruitment: *"CL-suspected patients were enrolled consecutively from February 2019 to December 2020:"* |
|  | *Test methods* | **10a** | Index test, in sufficient detail to allow replication | Methods section, subsection Design, population and recruitment: *"The performance of the Inbios CL Detect Rapid Test was assessed using a dental broach sample as well as a skin slit sample"*  Methods section, subsection Sample collection: *"Topical EMLA cream (5% lidocaine/prilocaine) was applied on the lesion for 30-60 minutes to limit patient discomfort. The dental broach sample was taken according to the supplied instructions on the part of the lesion that was assumed to be most active (predominantly on the border, and avoiding mucosal parts of the lesion where possible) [14]. The sample was placed in three drops of kit lysis buffer, and tissue was flushed from the dental broach using a pipet, after which the dental broach was discarded. The sample was further processed for the RDT and PCR (see below). Furthermore, two skin slits were taken on approximately the same lesion site as the dental broach sample.*  *The second skin slit was placed in a tube containing three drops of kit lysis buffer and tissue was flushed from the scalpel using a pipet, after which the scalpel was discarded."*  *"20 µL of the dental broach or skin slit sample in lysis buffer was immediately added on the test strip, after which it was placed in a tube containing 3 drops of chase buffer. The test was read and checked (blinded) after 20 minutes."* |
|  |  | **10b** | Reference standard, in sufficient detail to allow replication | Methods section, subsection Design, population and recruitment: *"… a combined reference test of skin slit microscopy and skin slit PCR. A patient was considered positive for the reference test if either skin slit microscopy or PCR was positive."*  *" The first skin slit was placed on a microscopy slide, smeared and stained for Giemsa as per routine practice. Results were red by two readers blinded to the other readers' microscopy result as well as the PCR results, and positive results were rated from +1 to +6. The second skin slit was placed in a tube containing three drops of kit lysis buffer and tissue was flushed from the scalpel using a pipet, after which the scalpel was discarded."*  See section Methods, subsection Molecular tests for the description of the PCR test. |
|  |  | **11** | Rationale for choosing the reference standard (if alternatives exist) |  |
|  |  | **12a** | Definition of and rationale for test positivity cut-offs or result categories  of the index test, distinguishing pre-specified from exploratory | NA, predefined by test kit. |
|  |  | **12b** | Definition of and rationale for test positivity cut-offs or result categories  of the reference standard, distinguishing pre-specified from exploratory | See section Methods, subsection Molecular tests for the description of the PCR test. |
|  |  | **13a** | Whether clinical information and reference standard results were available  to the performers/readers of the index test | See Methods, subsection Cl Detect Rapid Test: *"The test was read and checked (blinded to PCR and microscopy results) after 20 minutes."* |
|  |  | **13b** | Whether clinical information and index test results were available  to the assessors of the reference standard | Methods, subsection Sample collection: *"*[microscopy] *results were read by two readers blinded to the other readers' microscopy result as well as the PCR results (complete blinding to index test results could not be guaranteed)"*  Methods, subsection Molecular tests: *"PCR tests were done in batch, blinded to index test results."* |
|  | *Analysis* | **14** | Methods for estimating or comparing measures of diagnostic accuracy | Methods, subsection Data collection and analysis: *"Data analysis was done in R version 3.6.1. Numbers and proportions and medians and interquartile range (IQR) were used to describe the population. Sensitivity, specificity, as well as positive and negative predictive values with 95% confidence intervals were calculated for the CL Detect Rapid Test using the dental broach and the skin slit samples, against a combined reference of skin slit microscopy and PCR.*  *Subgroup analyses were done for the different CL types, and for patients with ulcerative lesions of less than four months duration (which is the population as recommended by the Inbios CL Detect Rapid Test manual) using Chi-square tests. McNemar's test was used to compare the sensitivity of the RDT on the skin slit to the dental broach."* |
|  |  | **15** | How indeterminate index test or reference standard results were handled | Methods, subsection Design, population and recruitment: "*Patients for whom the PCR result was invalid were not included in the diagnostic accuracy analysis."* |
|  |  | **16** | How missing data on the index test and reference standard were handled | There was no missing data of the index test and reference test |
|  |  | **17** | Any analyses of variability in diagnostic accuracy, distinguishing pre-specified from exploratory | Methods, subsection Data collection and analysis: " *Subgroup analyses were done for the different CL types, and for patients with ulcerative lesions of less than four months duration (which is the population as recommended by the Inbios CL Detect Rapid Test manual) using Chi-square tests. McNemar's test was used to compare the sensitivity of the RDT on the skin slit to the dental broach."* |
|  |  | **18** | Intended sample size and how it was determined | Methods, subsection Sample size: "*Our initial sample size calculation was done based on a precision of 10%, a power of 80% and an estimated sensitivity and specificity of the rapid test using the skin slit sample of 70%, which gave a sample size of 305 participants. After adding 15% due to expected loss to follow up, our initial sample size was 350 participants. Due to unavailability of the Inbios CL Detect Rapid Test since June 2020, we had to stop recruitment early. We analyzed the 165 recruited patients for whom this test was done in this report. "* |
|  | **RESULTS** |  |  |  |
|  | *Participants* | **19** | Flow of participants, using a diagram | See Supplementary Figures S1 and S2. |
|  |  | **20** | Baseline demographic and clinical characteristics of participants | See Table 1. |
|  |  | **21a** | Distribution of severity of disease in those with the target condition | See proportion of LCL, MCL, DCL in Table 1, and their characteristics indicating severity. |
|  |  | **21b** | Distribution of alternative diagnoses in those without the target condition | NA |
|  |  | **22** | Time interval and any clinical interventions between index test and reference standard | NA (sample collection was the same time) |
|  | *Test results* | **23** | Cross tabulation of the index test results (or their distribution)  by the results of the reference standard | See Table 2. |
|  |  | **24** | Estimates of diagnostic accuracy and their precision (such as 95% confidence intervals) | See Table 2. |
|  |  | **25** | Any adverse events from performing the index test or the reference standard | NA |
|  | **DISCUSSION** |  |  |  |
|  |  | **26** | Study limitations, including sources of potential bias, statistical uncertainty, and generalisability | See Discussion, last paragraph: *" this study is subject to several limitations. Since we encountered some contamination during DNA isolation, the PCR result was invalid for 11 samples as the sample values were too close to the value obtained for the contaminated negative extraction control. These samples are currently not included in the sensitivity and specificity analysis. If they were positive, this would lead to lowered sensitivity of the rapid tests, as all rapid tests (and microscopy results) of these patients were negative. If any of these cases were truly negatives, which is the most likely scenario, this does not affect the specificity estimates, although it causes the 95% confidence interval for these estimates to narrow. Another limitation is the relatively low number of negative patients, which means our specificity estimates are relatively inaccurate. Lastly, species typing is not included in this paper, but based on previous studies we expect all patients to be affected by L. aethiopica [8,28,29]. This will be verified in a follow-up study."* |
|  |  | **27** | Implications for practice, including the intended use and clinical role of the index test | Discussion, paragraph 4: "*Based on the limited sensitivity, we do not recommend the use of the Inbios CL Detect Rapid Test for routine use for the detection of L. aethiopica."* |
|  | **OTHER INFORMATION** |  |  |  |
|  |  | **28** | Registration number and name of registry | Methods, subsection Ethics statement: *"This study is registered at ClinicalTrials.gov as NCT03837431."* |
|  |  | **29** | Where the full study protocol can be accessed | NA |
|  |  | **30** | Sources of funding and other support; role of funders | See section Financial disclosure statement: "*This work was supported by the Directorate-General development cooperation and Humanitarian Aid (DGD), under the FA4 framework collaboration of the Institute of Tropical Medicine (Antwerp, Belgium) and the University of Gondar (Gondar, Ethiopia), granted to JvG and ED. The funders had no role in study design, data collection and analysis, decision to publish, or preparation of the manuscript*." |
|  |  |  |  |  |
