# Supplementary material for: Evaluation of the CL Detect Rapid Test in Ethiopian patients suspected for Cutaneous Leishmaniasis
Source: PLoS Negl Trop Dis. 2022 Jan 18;16(1):e0010143. doi: 10.1371/journal.pntd.0010143 (PMC8797207; doi:10.1371/journal.pntd.0010143)
Supplement: S2 Table — CI: Confidence interval; DB: dental broach; RDT: Cl Detect Rapid Test, SS: Skin slit. (DOCX) [file pntd.0010143.s006.docx]

|  | Cases, N=128 | | Non-cases, N=37 | | Diagnostic performance | | | |
| --- | --- | --- | --- | --- | --- | --- | --- | --- |
|  | Positive | Negative | Positive | Negative | Sensitivity (95% CI) | Specificity (95% CI) | PPV (95% CI) | NPV (95% CI) |
| Test |  |  |  |  |  |  |  |  |
| Microscopy | 71 | 57 | 0 | 37 | 55.5 (46.8 - 63.8) | 100 (90.6 - 100) | 100 (94.9 - 100) | 44.0 (33.9 - 54.7) |
| SS RDT | 40 | 88 | 1 | 36 | 31.3 (23.9 - 39.7) | 97.3 (86.2 - 99.5) | 97.6 (87.4 - 99.6) | 29.0 (21.8 - 37.6) |
| DB RDT | 29 | 99 | 1 | 36 | 22.7 (16.3 - 30.6) | 97.3 (86.2 - 99.5) | 96.7 (83.3 - 99.4) | 26.7 (20.0 - 34.7) |

**S2 Table. Sensitivity analysis for the different diagnostic tests with re-classification of invalid test results**

CI: Confidence interval; DB: dental broach; RDT: Cl Detect Rapid Test; NPV: negative predictive value; PPV: positive predictive value; SS: Skin slit
